# Supplementary material for: A Fresh Perspective on Cyanobacterial Paralytic Shellfish Poisoning Toxins: History, Methodology, and Toxicology
Source: Mar Drugs. 2025 Jun 27;23(7):271. doi: 10.3390/md23070271 (PMC12301064; doi:10.3390/md23070271)
Supplement: Supplementary file 1 [file marinedrugs-23-00271-s001.zip › marinedrugs-3651067-supplementary.pdf]

Supplementary Material

Title: A Fresh Perspective on Cyanobacterial Paralytic Shellfish Poisoning Toxins: History, Methodology, and Toxicology

Zacharias J. Smith[1], Kandis M. Arlinghaus[2], Gregory L. Boyer[3], Cathleen J. Hapeman[1]

[1]US Department of Agriculture, Agricultural Research Service, Hydrology and Remote Sensing Laboratory, 10300 Baltimore Ave, Beltsville, MD 20705

[2]Oak Ridge Institute for Science and Education (ORISE) Fellow to the National Oceanic and Atmospheric Administration, National Ocean Service, 331 Fort Johnson Road, Charleston, South Carolina 29412

[3]State University of New York College of Environmental Science and Forestry, Department of Environmental Biology, 1 Forestry Drive, Syracuse, New York 13210

*Corresponding author: Zacharias J. Smith (Zach.Smith@usda.gov)

Supplemental Information

Table S1. Reproduction of Table 1-1 from the 2000 dissertation of Hideyuki Onodera, with the permission of the author, showing the types and number of cyanobacterial strains measured for PSPTs. A partial version of this table is presented in a conference paper, which is citation 81 in the review.

Table 1-1. Cyanobacterial samples surveyed of the production of paralytic shellfish toxins by the new screening method

#	species	code	collection		#	species	code	collection		#	species	code	collection	
1	***Anabaenopsis* sp.**	A1	Aug. '90	Yamagata	23	***M. aeruginosa***	M108-1	'77	Tokyo	45	***An. lemmermannii***	JUL2	Jul. '93	"
2	***An. affinis***	AAf	Apr. '97	Tokyo	24	"	TAC13	Jan. '78	Ibaraki	46	"	PH-47	Jul. '93	"
3	***An. planktonica***	A5	"	"	25	"	TAC60	Sep. '84	Nagano	47	***An.* cf. *cylindrica***	PH-133	'94	"
4	***An. reniformis***	A20	Sep. '90	Hyogo	26	"	TAC66	Sep. '84	"	48	***An. spiroides***	AS	–	Brazil
5	"	A21	"	"	27	"	TAC80	Sep. '84	Fukui	49	***Anabaena* spp.**	JUN27	Jun. '94	Aarhus, Denmark
6	"	A22	"	"	28	"	TAC113	Oct. '88	Ehime	50	***Ap. flos-aquae***	NH-5S1	Mar. '91	NH, USA
7	***An. viguieri***	A23	"	"	29	"	TAC91	Aug. '84	Hokkaido	51	"	NH-5S2	Jun. '91	"
8	***An. mendotae***	A43	Jun. '91	Ibaraki	30	***M. viridis***	TAC44	Jan. '78	Ibaraki	52	"	LMECYA31	Jun. '96	Montargil, Portugal
9	***An. heterospora***	A47	"	Tochigi	31	"	TAC95	Aug. '84	Hokkaido	53	***C. raciborskii***	T1	'94	Sao Paulo, Brazil
10	***An. teneticaulis***	HA1	Feb. '92	Tokyo	32	"	TAC64	Sep. '84	Nagano	54	"	T2	'96	"
11	"	HA2	"	"	33	***M. wesenbergii***	TAC85	Sep. '84	Shimane	55	"	T3	'96	"
12	"	HA8	"	"	34	***Microcystis* sp.**	TOK	Oct. '95	Tokushima	56	"	BH	–	Minas Gerais, Brazil
13	***Anabaena* sp.**	NO2	Aug. '96	Miyagi	35	"	NO1	Aug. '96	Miyagi	57	"	ING	–	Pernambuco, Brazil
14	"	NO3	"	"	36	***O. mougeotii***	KO2	Jul. '92	Ibaraki	58	"	TUP	–	"
15	"	NO5	"	"	37	"	KO3	"	"	59	***Lyngbya wollei***	OSS#2	Jul. '93	Alabama, USA
16	"	A51	Jul. '91	Hokkaido	38	"	KO4	"	"	60	"	SB#3	Jan. '94	"
17	"	AA	Nov. '95	Shiga	39	"	KO5	"	"	61	***Lyngbya* sp.**	Q8Ly1	Sep. '93	Quidon, China
18	***Ap. issatschenkoi***	Aph5	Feb. '92	Ibaraki	40	***An. circinalis***	MB01	Feb. '93	SA, Australia	62	"	Q8Ly2-1	"	"
19	***Lyngbya* sp.**	TM2-10	Jul. '86	Tokyo	41	"	BU02	Nov. '93	NSW, Australia	63	"	Q8Ly2-2	"	"
20	***M. aeruginosa***	M228	Jul. '77	Nagano	42	"	TA04	Jan. '93	VC, Australia	64	"	Korea	–	Korea
21	"	M203-2	"	"	43	***An. flos-aqaue***	BC	–	Portugal	65	***Oscillatoria* sp.**	SUM95	'95	Denmark
22	"	M230	"	"	44	***An. lemmermannii***	JUN28	Jun. '94	Aarhus, Denmark	66	***Planktothrix* sp.***	FP1	Aug. '97	Varese, Italy

An. : *Anabaena*, *Ap.* : *Aphanizomenon*, *C.* : *Cylindrospermopsis*, *M.* : *Microcystis*, *O.* : *Oscillatoria*

SA : South Australia; NSW : New South Wales; VC : Victoria; NH : New Hampshire

* Natural bloom sample

Table S2. Reproduction of Table S3.3 from the 2019 dissertation of Zacharias Smith (citation 4) showing the variability in sensitivity/limit of detection for different LC-MS/MS methods.

Table S3.3. LODs for each of the LC-MS/MS methods reported in fmol of PSTs on column based on the injection volumes for the respective analytical method.

Toxin	LC-MS/MS Method 1 LODs	LC-MS/MS Method 2 LODs	LC-HRMS Method 3 LODs
STX	35.6	11	40
NEO	37.9	80	170
GTX1	88.9	150	41
GTX2	150.5	100	42
GTX3	41.1	97.5	15
GTX4	18.5	150	99
GTX5	24.6	110	
GTX6		170	
dcSTX	181.3	19.25	160
dcNEO	78.5	95	
dcGTX1		475	
dcGTX2	466.4	232.5	27
dcGTX3	51.7	85	
dcGTX4		325	
C1	38.3	212.5	
C2	37.2	62.5	
C3		350	
C4		375	
M1			
M2			
M3			
M4			
M5			
doSTX		21.5	
LWTX1			
LWTX2			
LWTX3			
LWTX4			
LWTX5			
LWTX6			
11-OH-dcSTX			
12,12dido-dcSTX			
12-deoxy-doSTX			
12-deoxy-dcSTX			

Table S3. Reproduction of Table 2-4 from the 1980 dissertation of Gregory Boyer (citation 41) showing the mouse bioassay toxicity in MU/mg for several 11-OH derivatives of saxitoxin and decarbamoylsaxitoxin.

TABLE 2-4

Chromatographic Properties of 11-(OSO_3^-)STX and Its Derivatives

Compound	Specific activity (MU/mg)	TLC R_f[a] PEAW solvent	EAH solvent	Electrophoretic mobility (R_{STX})
11-(OSO_3^-)STX	2300 ± 300	0.71/0.66[b]	0.60	0.55
11-(OSO_3^-)STXOH	< 200	0.58	0.52	0.55
11-(OH)STX	≈ 2300	0.31	0.35	0.97
11-(OH)STXOH	< 200	0.18	0.38	0.98
11-(OH)dcSTX	900	0.11	0.05	0.95
STX[c]	5500 ± 500	0.55	0.50	1.00
dcSTX[c]	3700 ± 400	0.47	0.50	1.05

[a] Silica Gel G, E. Merck without fluorescent indicator. Solvents: PEAW, pyridine:ethyl acetate: acetic acid:water, 75:25:15:30; EAH, ethyl acetate:acetic acid:water, 60:15:25.

[b] R_f 0.66 is due to presence of the minor C-11 epimer (section 2-C).

[c] Included for comparison.
